# RenseNet: A Deep Learning Network Incorporating Residual and Dense Blocks with Edge Conservative Module to Improve Small-Lesion Classification and Model Interpretation

**DOI:** 10.3390/cancers16030570

**Published:** 2024-01-29

**Authors:** Hyunseok Seo, Seokjun Lee, Sojin Yun, Saebom Leem, Seohee So, Deok Hyun Han

**Affiliations:** 1 Bionics Research Center, Biomedical Research Division, Korea Institute of Science and Technology (KIST), Seoul 02792, Republic of Korea; ykykyk112@kist.re.kr (S.L.); yunsojin98@gmail.com (S.Y.); toqha1215@sogang.ac.kr (S.L.); soseohee1226@gmail.com (S.S.); 2 Department of Urology, Samsung Medical Center (SMC), Seoul 06351, Republic of Korea; deokhyun.han@samsung.com

**Keywords:** classification, deep learning, explainable AI, heatmap, small lesion

## Abstract

**Simple Summary:**

Small-target classification in an image is still challenging in spite of emerging deep learning-based techniques. This study focused on the development of deep learning models for small-lesion classification. The proposed Rense block and edge conservative module enables deep learning models to extract better features of small lesions in images. Our RenseNet was validated with quantitative classification accuracy and a qualitative explanation heatmap for kidney stone and lung tumor computed tomography (CT) image datasets.

**Abstract:**

Deep learning has become an essential tool in medical image analysis owing to its remarkable performance. Target classification and model interpretability are key applications of deep learning in medical image analysis, and hence many deep learning-based algorithms have emerged. Many existing deep learning-based algorithms include pooling operations, which are a type of subsampling used to enlarge the receptive field. However, pooling operations degrade the image details in terms of signal processing theory, which is significantly sensitive to small objects in an image. Therefore, in this study, we designed a Rense block and edge conservative module to effectively manipulate previous feature information in the feed-forward learning process. Specifically, a Rense block, an optimal design that incorporates skip connections of residual and dense blocks, was demonstrated through mathematical analysis. Furthermore, we avoid blurring of the features in the pooling operation through a compensation path in the edge conservative module. Two independent CT datasets of kidney stones and lung tumors, in which small lesions are often included in the images, were used to verify the proposed RenseNet. The results of the classification and explanation heatmaps show that the proposed RenseNet provides the best inference and interpretation compared to current state-of-the-art methods. The proposed RenseNet can significantly contribute to efficient diagnosis and treatment because it is effective for small lesions that might be misclassified or misinterpreted.

## 1. Introduction

The success of deep learning (DL) in computer vision has led to the increased application of artificial intelligence (AI) in medical image analysis [1,2]. DL-based AI models have been applied to clinical vision applications, such as lesion detection, classification, and segmentation, thereby outperforming conventional machine learning models [3,4,5,6]. Moreover, explainable AI (XAI) allows users to interpret and trust the results predicted by the AI model [7]. Clinical vision applications often require high reliability; therefore, there might be limitations in directly applying DL models in real practice, as the internal process of DL models is a black box type. XAI, an emerging technique in the field of vision, helps to build trust and confidence in model prediction, which can lead to a wider application of DL in the clinical field. Gradient-weighted class activation mapping (Grad-CAM) is the principal XAI algorithm for comprehending the prediction results without modifying the model [8]. Grad-CAM is a generalization of the class activation map (CAM) [9], and recent advanced techniques such as Grad-CAM++ [10], Ablation-CAM [11], and XGrad-CAM [12] are also based on Grad-CAM. Layer-wise relevance propagation (LRP) has been introduced as another method to explain model decisions [13]. However, the explanations are different across DL models, even though the same dataset and XAI algorithm are applied [14]. Therefore, there is room to improve the explanation of results using a DL model design.

Accurate classification of small lesions in medical images for early diagnosis and efficient treatment leads to more effective recovery; however, small-object classification is a challenging and important problem in computer vision research. In general, small objects often exhibit non-evident visual features. Previous studies [3,4] showed that the detection and segmentation accuracy of small lesions is not comparable to that of large targets, despite using DL techniques. This is because information loss of small lesions owing to subsampling (e.g., pooling) is crucial. To overcome the limitations, those studies, which focused on the segmentation task, enhanced performance of a U-Net-based structure by introducing adaptive loss function and task-consistency regularization schemes. In terms of XAI, the natural image dataset (for example, the ImageNet dataset) includes evident and bulky objects; therefore, the heatmap result of Grad-CAM from the DL-based classification model is consistent with human cognition. However, in the medical image dataset, there are many small-lesion scenarios, such as kidney stones, tumors, and blood vessels. The heatmaps estimated from XAI algorithms for small-lesion classification do not provide a reasonable interpretation at times because the prediction results for classification are not propagated backward well in the DL model owing to information loss in the model. Hence, existing DL models exhibit the problem of misprediction and misinterpretation in small-target tasks, which hinders their common use in clinical sites.

In this study, we propose a deep-learning model to improve the classification of small lesions. Our model generates a more reliable explanation heatmap in comparison with state-of-the-art models. The key contributions of this study are as follows.

- A backbone network, which is composed of Rense blocks merged with residual and dense blocks, was proposed using mathematical analysis such that we can utilize the skip connections more efficiently and provide an insight for model structures.
- The edge conservative module was designed in the compensation paths, by preserving the image detail lost from the subsampling (pooling) operation. Auxiliary features were then extracted in this module for better inference.
- The performance of the proposed model was evaluated using a public CT dataset of lung tumors and a private CT dataset of kidney stones, where there are often small-lesion scenarios. Validation results in terms of classification and Grad-CAM-based heatmaps show that the proposed model classifies small lesions better and can provide more trust to clinical users in their decision-making compared with cutting-edge techniques.

## 2. Related Works

Object classification is a basic task in the field of computer vision. With the development of machine learning, object classification in medical images has also advanced. Before DL was receiving significant attention, classical machine learning algorithms were applied to medical images. Machine learning techniques often require handcrafted features (or manually extracted features), which rely on human intervention, to build a mathematical model in a data-driven manner [15]. For feature engineering in medical image analysis, Haralick features calculated by a gray-level co-occurrence matrix of neighboring gray levels (GLCM) in the image were introduced and compared with other feature descriptors—local binary patterns (LBP), gray-level run-length method (GLRLM), and gray-level difference method (GLDM)—which are well-known statistical texture-based feature descriptors [16]. The auto-correlation Gabor feature (AGF), which is invariant to scale, rotation, and illumination changes in an image, was applied to gastroenterology images [17]. Further, scale-invariant feature transform (SIFT) feature-based representations have achieved gains [18,19]. Over the last few years, aggressive investigations using DL have shifted the paradigm, and convolutional neural networks (CNNs) have been widely applied to object classification with big success. The basic modules of CNNs, such as inception [20], residual [21], and dense [22] blocks, were developed. Recently, EfficientNet [23], which proposed compound scaling for model depth, width, and resolution, and a vision transformer (ViT) [24], which adopted the transformer in natural language processing to the vision field, have been applied to biomedical images [25,26]. In the meantime, various deep learning models that use and combine residual and dense blocks have been studied [27,28]; however, most of them are employed empirically. As such, in this paper, we provide the mathematical analysis and insight for the optimal combination of residual and dense blocks instead of relying on empirical results.

## 3. Materials and Methods

**Figure 1:**
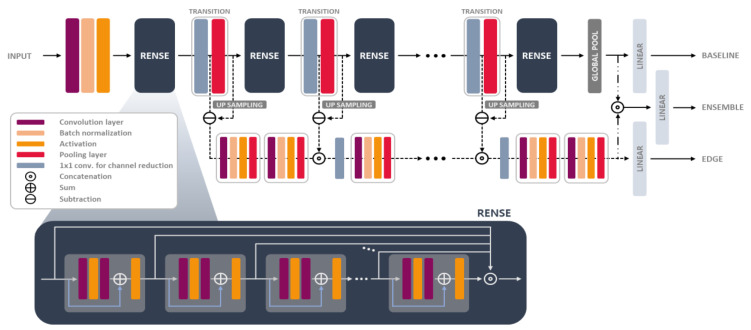
Schematic diagram of the proposed RenseNet (Rense blocks with an edge conservative module).

A full schematic of the proposed network composed of Rense blocks and edge conservative modules is shown in Figure 1. The remainder of this section includes a detailed mathematical and theoretical description of the proposed model.

### 3.1. Residual and Dense Blocks

**Figure 2:**
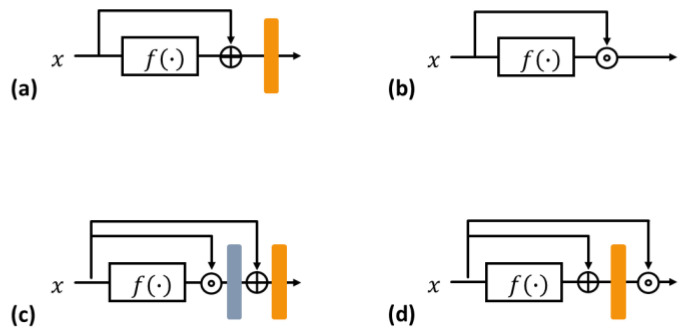
Simplification of basic blocks. (**a**) Residual block; (**b**) dense block; (**c**) switched Rense (sRense) block—dense block inside a residual block; and (**d**) Rense block—residual block inside a dense block (orange layer: activation, gray layer: 1 × 1 convolution for channel reduction).

Currently, deep neural network (DNN) models often use skip connections to increase learning efficiency. A residual block is defined by a skip connection that learns the residual functions concerning the layer input signal. Then, the main path and skip connection are added before applying nonlinear activation [21]. To easily provide insight, the 2D residual block can be simplified, as shown in Figure 2a. The output of the *n*th residual block is expressed as:

(1) $$y=A\left\{f\left(x\right)+x\right\}\in {ℜ}^{W_{n}\times H_{n}\times C_{n}},\;\mathrm{where}\;x\in {ℜ}^{W_{n}\times H_{n}\times C_{n}}.$$

In Equation (1), A(·) is the activation operator and f(·) is a function that includes the convolution, activation, and normalization schemes, where $f:{ℜ}^{W_{n}\times H_{n}\times C_{n}}\to {ℜ}^{W_{n}\times H_{n}\times C_{n}}$. A dense block also employs skip connections to accumulate layer input signal along the channel direction. Then, the *n*th dense block with skip connection [1] in Figure 2b results in:

(2) $$y=f\left(x\right)⊚x\in {ℜ}^{W_{n}\times H_{n}\times 2C_{n}},$$

where ⊚ denotes the concatenation along the channel direction. The residual block effectively reduces the search range in the feature space because it learns only residual signals instead of full output signals. A dense block flexibly utilizes previous features because of their accumulation. Basically, the model can avoid the gradient vanishing problem by skip connections in both blocks such that gradients propagate backward well.

### 3.2. Rense Block

**Figure 3:**
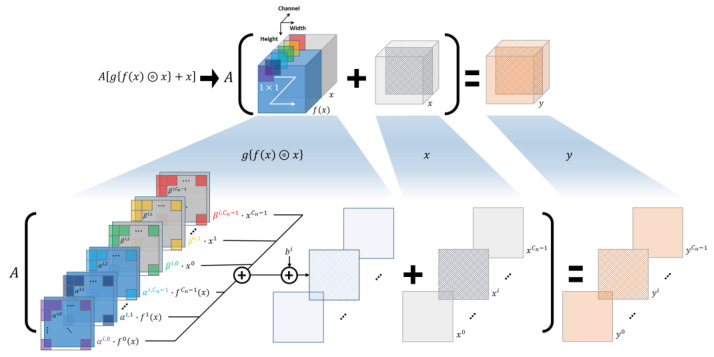
Visualization of mathematical analysis for sRense block. This graphical detail shows linear decomposition of Equation (3), which turns into Equation (4).

To take advantage of both blocks, two cases were considered, as shown in Figure 2c,d. Figure 2c shows the case where the dense block is inside the residual block. The output of this block is calculated as follows:

(3) $$y=A\left[g\left\{f\left(x\right)⊚x\right\}+x\right]\in {ℜ}^{W_{n}\times H_{n}\times C_{n}},$$

where g(∙) is the 1 × 1 convolution operator used to reduce the dimension of the channels. A 1 × 1 convolution generates a channel-wise weighted sum; therefore, concatenation and a 1 × 1 convolution can be demonstrated with linear operations, as shown in Figure 3. In other words, the element of y in the *i*th channel location, that is, the output feature map in the *i*th channel, is reformulated as

(4) $$y^{i}=A\left[\sum_{k=0}^{C_{n}-1}\left\{\alpha^{i,k}f^{k}\left(x\right)+\beta^{i,k}x^{k}\right\}+b^{i}+x^{i}\right],\;=A\left\{\sum_{k=0}^{C_{n}-1}\alpha^{i,k}f^{k}\left(x\right)+\sum_{k=0}^{C_{n}-1}(\beta^{i,k}+\delta^{i,k})x^{k}+b^{i}\right\}\in {ℜ}^{W_{n}\times H_{n}}.$$

Here, $f^{k}\left(x\right)\in {ℜ}^{W_{n}\times H_{n}}$ and $x^{k}\in {ℜ}^{W_{n}\times H_{n}}$ are the elements of $f\left(x\right)$ and x at the *k*^th^ channel location, respectively; $b^{i}$ is the aggregated bias term of all 1 × 1 convolutions for the *i*th channel location of the output; and $\delta^{i,k}=1$ when k=i; otherwise, $\delta^{i,k}=0$. $\alpha^{i,k}$ and $\beta^{i,k}$ are the kernel weights of $f^{k}\left(x\right)$ and $x^{k}$ for the output element of y in the *i*th channel location, respectively. Consequently, this block can generalize the residual block in Equation (1) because an element of y in the *i*th channel location of Equation (1) can be rewritten with that of Equation (4), as follows:

(5) $$y^{i}=A\left\{f^{i}\;\left(x\right)+x^{i}\right\}\in {ℜ}^{W_{n}\times H_{n}},$$

where both $\alpha^{i,k}=1$ and $\beta^{i,k}=1$ when k=i; otherwise, $\alpha^{i,k}=0$ and $\beta^{i,k}=0$. Here, the additional bias term $b^{i}$, which can shift the activation function, equals zero. Thus, the original residual block in Figure 2a is a special case of a dense block inside the residual block in Figure 2c because the dense block inside the residual block does not exhibit any limitations for $\alpha^{i,k}$, $\beta^{i,k}$, or $b^{i}$. Subsequently, this block can obtain a gain from the flexibility of the residual learning, but the original dense effect does not move the needle significantly. In this study, it is defined as a switched Rense (sRense) block.

Equation (5) cannot be further expanded by other linear operations, so we can expect the full effects from addition and concatenation of identity in Equation (5). In other words, the respective skip connections in the residual and dense blocks have their original power, and they are clearly formulated into the sum and concatenation of the input, that is, this block learns the residual function and simultaneously retains the previous features. This block is called Rense, and is the basic block of the proposed RenseNet.

### 3.3. Edge Conservative Module

Generally, most DNNs comprise a series of layers, including convolution, activation, and pooling operations. The pooling operation is a type of subsampling scheme used to overcome the small receptive field in the convolution kernels. Otherwise, the inference of the model is biased toward the local features of the input data. To extract the global features effectively, the feature maps are resized in two ways—pooling operations and stride factor—in convolution, as follows:

(6) $$D:{ℜ}^{W_{n}\times H_{n}\times C_{n}}\to {ℜ}^{W_{n}/S_{n}\times H_{n}/S_{n}\times C_{n}},$$

where D is a subsampling operator; $S_{n}$, $C_{n}$, $W_{n}/S_{n}$, and $H_{n}/S_{n}$ are integers; and $W_{n}$, $H_{n}$, and $C_{n}$ represent the width, height, and channel dimensions in the *n*th layer, respectively. After the subsampling operation in the *n*th layer, the size of the feature maps in the spatial dimension is reduced by a factor of $S_{n}$ in Equation (6), which causes information loss in the original pixel grids and results in blurring: Blurring, owing to max pooling, is also explained in terms of the frequency domain. Subsampling generates aliasing artifacts in the frequency domain [29]. Specifically, max pooling, which is commonly applied in CNNs, is a type of incoherent (pseudo-random) sampling. Its filter does not have a predefined shape because sampling within a 2 × 2 window depends on the input signals. Inspired by compressed-sensing theory, incoherent sampling causes noise-like aliasing artifacts (incoherent interferences) in the frequency domain [30]. Subsequently, noise-like aliased signals are mixed with the original frequency components. In general images, the energy of the high-frequency component is basically lower than that of the low-frequency component; therefore, the high-frequency component is more sensitive to aliasing perturbation, which degrades the image details.

**Figure 4:**
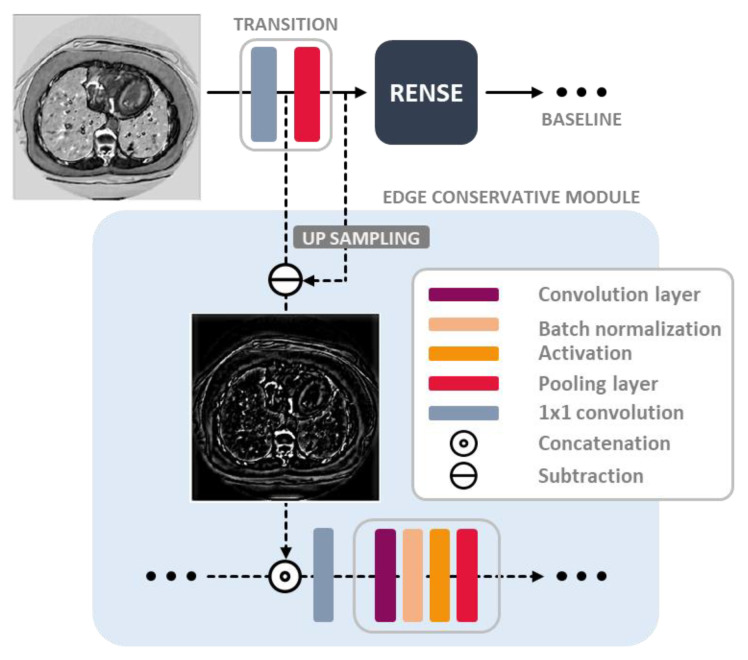
An example of the feature maps extracted by the baseline path and the edge conservative module.

In the computer vision problem, blurring confuses object recognition, and it is a more crucial issue in small object classification because pixels in a small object might merge into neighboring pixels and easily lose their original shape. Therefore, to fully utilize the structural information of the object, we proposed an edge conservative module, as shown in Figure 1 and Figure 4. The degradation of the image detail is compensated by a combination of the residual path and upsampling after the pooling operation, as shown in Figure 4. In the edge conservative module, all compensated features are incorporated into another CNN path to extract auxiliary features. Here, a shallow structure was applied to avoid overfitting because the auxiliary features were sparse.

### 3.4. RenseNet Details

Our network had two parallel forward paths, as shown in Figure 1: first, the baseline path composed of the Rense blocks in Figure 2d, where the other configurations are the same as those of ResNet50 [21], but all residual blocks are replaced by the proposed Rense blocks and there are transitions before pooling layers; and second, an edge conservative path that has eight 3 × 3 convolution layers and three 1 × 1 layers. Three output layers, then, were used to calculate the classification scores after linear projections (fully connected layers) in this module. The cross-entropy (CE) loss was adopted, and the weights for each CE loss were 1:0.3:0.3 for the baseline, ensemble, and edge conservative paths, respectively. They were empirically set, and the batch size was set to eight. All learning processes were stopped at 150 epochs owing to performance saturation and computation time.

### 3.5. Dataset and Preparation

In this study, the proposed model was verified using two datasets. The first dataset was composed of CT images obtained from patients with kidney stones. This dataset was acquired from the Samsung Medical Center (SMC), Seoul, South Korea, with Institutional Review Board approval (IRB 2021-04-113). There were 60 patient scans and 1468 slice images (positive stone cases 367 slices and negative cases 1101 slices). Each slice image was 512 × 512, and annotations were made by urologists, radiologists, and clinical experts to determine the presence of kidney stones in the CT images. The dataset was separated into 1236 images of training (positive 309, negative 927) and 232 images of validation (positive 58, negative 174) for fivefold cross-validation. In this dataset, there were only classification labels; therefore, quantitative comparisons were applied to the classification accuracy. The second dataset consisted of CT images of lung cancer tumors. These data were from the Decathlon Challenge [31] in the public domain and included 60 scans with 512 × 512 slice images. There were a total of 3314 slice images (positive tumor cases 1657 slices and negative tumor cases 1657 slices). Specifically, 2814 training images (positive 1407, negative 1407) and 500 validation images (positive 250, negative 250) were prepared for fivefold validation. This public dataset provided segmentation labels such that we could evaluate both classification and explanation accuracy. In our experiments, all data separation for training and validation complied with the patient-level splits. In the training process, random cropping, flip, rotation, and translation were applied for data augmentation.

### 3.6. Grad-CAM-Based Heatmap

To validate the interpretability, heatmaps were estimated by the Grad-CAM algorithm [9] at the final feature extraction layer of all models. The Grad-CAM results were binarized over an 80% threshold. The public lung dataset provides segmentation labels. Thus, heatmaps can be evaluated using the exact shape and location of the target. The dice similarity coefficient (DSC) between the masks generated by the Grad-CAM-based heatmap and segmentation labels was calculated. Thus, we checked that the models examined the target lesion when they made a decision.

## 4. Results

### 4.1. Kidney Stone Dataset

The classification accuracy, precision, recall, specificity, and F1 score for the kidney stone dataset are presented in Table 1, where they are compared against seven models. Their 95% confidence intervals are calculated in Table 2. The proposed RenseNet stands for Rense block with an edge conservative module (W/E).

First, we ensured the effectiveness of the edge conservation module. Most metrics showed improved scores when combined with the edge conservative module (W/E). The maximum gains were 9.05, 26.81, 8.62, 9.20, and 16.60% for accuracy, precision, sensitivity, specificity, and F1 score, respectively. In terms of the basic block, the network based on Rense blocks, instead of sRense blocks, provided the best scores in accuracy, sensitivity, specificity, and F1. The F1 score indicates that the proposed RenseNet (Rense blocks + W/E) has the highest performance in harmonic of precision and sensitivity. A vision transformer (ViT) [24] where pretrained parameters are applied inferred biased results for specificity. The guided attention inference network (GAIN) [32], which was devised for weakly supervised detection with classification labels and general performance improvement, was not effective for small-lesion classification. We conducted *t*-tests under the null hypothesis H_0_: $\mu_{Rense\;block}=\mu_{m}$, where m stands for the compared methods with respect to metrics and μ the mean values in Table 1. We can reject H_0_ at the significance level 0.05 because all *p*-values are less than 0.05.

### 4.2. Lung Tumor Dataset

Similarly, the classification accuracy, precision, recall, specificity, and F1 score for the lung tumor dataset are presented in Table 3. Their 95% confidence intervals are calculated in Table 4.

The trend for the quantitative results with the lung tumor dataset was similar to that with the kidney stone dataset. The edge conservative module improved the scores in most cases.

The maximum gains were 8.92, 8.82, 10.43, 32.39, and 8.47% for accuracy, precision, sensitivity, specificity, and F1 score, respectively. RenseNet (Rense block with an edge conservative module) showed the best scores in terms of accuracy, precision, specificity, and F1 score. Here, ViT and GAIN were not powerful for lung tumor datasets. From the *t*-test, all *p*-values are found to be less than 0.05, so score differences in Table 3 are statistically meaningful.

**Figure 5:**
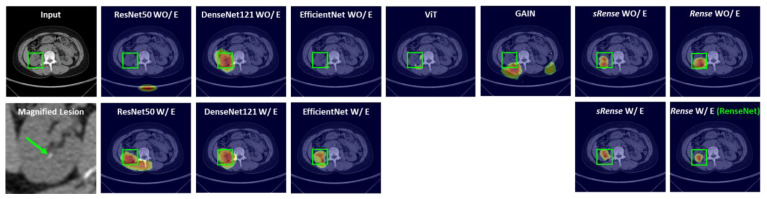
Graphical results of explainable heatmaps in kidney stone cases.

**Figure 6:**
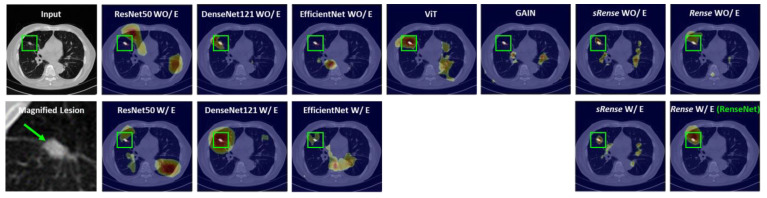
Graphical results of explainable heatmaps in lung tumor cases.

We can quantify the interpretability of the model because the lung dataset provided segmentation labels. From the DSC scores in Table 3, the tendency is similar to results of the other metrics in Table 1 and Table 3. The network composed of Rense blocks outperformed other networks, and an edge conservative module enhanced interpretability. Specifically, the network combined with the Rense block and an edge conservative module (RenseNet) exhibited the best performance. Graphical results for the explainable heatmaps are presented in Figure 5 and Figure 6. For the kidney stone dataset (Figure 5), most models focused on the kidney stone in the CT images, except ResNet50 WO/E, EffieicntNet WO/E, ViT, and GAIN.

However, RenseNet had a more refined and localized heatmap. Heatmaps from the lung dataset accurately indicated the lung tumors in DenseNet121 W/E and the proposed RenseNet. Moreover, RenseNet provided the best localized heatmap, as shown in Figure 6. The visual results of the explainable heatmaps are well aligned with the DSC results in Table 3. Therefore, the Rense block and an edge conservative module contributed to the accurate interpretability of the model. Furthermore, the computational cost for each model was compared. Table 5 implies RenseNet is economical and that there is not a big difference from the best, as transition layers effectively reduce feature size along the channel dimension.

## 5. Discussion

Many DL models are used to classify targets in images. However, most models are designed for large and evident target objects, for example, the ImageNet dataset. In medical images, the size of the lesions varies, and the boundaries might not be evident [33]. Therefore, it is difficult to match the performance of a model dedicated to large objects with medical image applications. Furthermore, the clinical purposes of DL models require greater reliability and confidence. Thus, XAI can be used to break the bottleneck for widespread DL in real clinical practice. Several algorithms have been developed, and Grad-CAM has become one of the principal algorithms for explaining model decisions. Despite its intuitive analysis for model inference, the explanations from Grad-CAM as well as other XAI algorithms often rely on DL models. Furthermore, model accuracy and interpretability for small lesions might not be appropriate, because most DL models lose their spatial information during inference, which is critical for small-lesion objects [3,33].

To improve both model accuracy and interpretability, we propose a Rense block with an edge conservative module that allows flexible use of skip connection paths in residual and dense blocks, and it compensates for spatial information lost in pooling layers. Through the mathematical analysis of skip connections, we found a way to effectively utilize previous features in feed-forward learning. The compensation module for the edge can prevent distortion or damage to small-lesion features. Basically, the Rense block and an edge conservative module allow the model to fully use information about small lesions.

The overall scores in the kidney stone dataset were better than those in the lung tumor dataset. Kidney stones have brighter HU (Hounsfield unit) than other tissues, which makes the inference easier. The results from both the kidney stone and lung tumor dataset show that RenseNet (Rense blocks with an edge conservative module) performed the best. Specifically, the sRense block is not as powerful as the Rense block, because the sRense block is a generalization of the residual block. We can expect a gain from the flexibility of residual learning, but it weakens the effect of dense learning. However, the Rense block has the merits of both residual and dense blocks, as formulated in Equation (6). This can be observed in the results of the Grad-CAM-based heatmap (Figure 5 and Figure 6) as well. GAIN [32] is the algorithm to refine the result of a Grad-Cam-based heatmap, which can be applied to weakly supervised detection. However, it does not work well in small lesions, because GAIN has a process that hides pixels relevant to the attention regions contributing to classification decision as complete as possible. For small lesions, attention regions are basically small in the heatmap and there are still enough areas not hidden in the image that the model can look over for inference. Thus, the model might be biased if large regions in the image that are not hidden provide other misinformation to the model, as shown in Table 1 and Table 3. The low performance of the ViT can be attributed to the pretrained model that does not describe small lesions well.

## 6. Conclusions

The proposed network aims to achieve high classification accuracy and reliable explanation of model decisions for small lesions in medical images. The Rense block and edge conservative module satisfied the main goal of this study. The experimental results presented in this study demonstrate the accuracy and reliability of the proposed RenseNet for small lesion lesions. Therefore, the proposed network provides new opportunities for clinical applications. In our network, we introduce an optimized integration of dense and residual blocks; however, we have not yet attained the optimal number of Rense blocks. Future research will focus on determining the ideal number of Rense blocks for improved computational efficiency and analyzing the target lesion size that can be effectively classified by our RenseNet.

## Figures and Tables

**Table 1:** Quantitative comparisons for kidney stone dataset. Here, WO/E and W/E are without and with the edge conservative module, respectively. Our RenseNet is the combined Rense block with edge conservative module.

Kidney Stone								
Metrics		ResNet50	DenseNet121	EfficientNet [23]	ViT [24]	GAIN [32]	sRense Block	Rense Block
Accuracy	WO/E	96.98	95.26	95.69	75.86	70.37	83.19	97.80
	W/E	97.41	96.98	96.12	-	-	92.24	97.84
Precision	WO/E	96.36	91.23	91.38	75.00	39.98	66.67	96.41
	W/E	96.43	98.11	98.04	-	-	93.48	96.49
Sensitivity	WO/E	91.38	89.53	91.38	5.17	81.25	65.52	94.75
	W/E	93.10	89.66	86.21	-	-	74.14	94.83
Specificity	WO/E	96.82	97.09	97.05	95.37	85.94	89.08	97.13
	W/E	98.86	99.41	98.85	-	-	98.28	99.43
F1 score	WO/E	93.80	90.37	91.38	9.67	53.59	66.09	95.57
	W/E	94.74	93.69	91.75	-	-	82.69	95.65

**Table 2:** 95% confidence intervals for kidney stone dataset. Here, WO/E and W/E are without and with the edge conservative module, respectively. Our RenseNet is the combined Rense block with edge conservative module.

Kidney Stone								
Metrics		ResNet50	DenseNet121	EfficientNet [23]	ViT [24]	GAIN [32]	sRense Block	Rense Block
Accuracy	WO/E	0.0061	0.0004	0.0102	0.0037	0.0032	0.0023	0.0127
	W/E	0.0069	0.0108	0.0037			0.0048	0.0153
Precision	WO/E	0.0007	0.0158	0.0094	0.0136	0.0157	0.0076	0.0057
	W/E	0.0052	0.0057	0.0064			0.0053	0.0071
Sensitivity	WO/E	0.0036	0.0069	0.0073	0.0037	0.0035	0.0058	0.0027
	W/E	0.0032	0.0115	0.0161			0.0014	0.0024
Specificity	WO/E	0.0128	0.0111	0.0090	0.0104	0.0127	0.0140	0.0090
	W/E	0.0142	0.0110	0.0086			0.0167	0.0029
F1 score	WO/E	0.0037	0.0045	0.0119	0.0065	0.0146	0.0067	0.0071
	W/E	0.0145	0.0087	0.0064			0.0003	0.0039

**Table 3:** Quantitative comparisons for lung stone dataset. Here, WO/E and W/E are without and with the edge conservative module, respectively. Our RenseNet is the combined Rense block with edge conservative module.

Lung Tumor								
Metrics		ResNet50	DenseNet121	EfficientNet [23]	ViT [24]	GAIN [32]	sRense Block	RENSE Block
Accuracy	WO/E	66.67	76.98	68.45	71.63	65.43	71.43	77.01
	W/E	75.59	77.58	73.61	-	-	73.41	79.58
Precision	WO/E	71.72	71.04	82.31	65.37	20.81	69.37	76.73
	W/E	80.54	79.39	84.68	-	-	70.69	84.97
Sensitivity	WO/E	56.89	91.23	48.41	91.40	90.58	78.87	83.55
	W/E	58.41	84.00	58.84	-	-	82.04	84.23
Specificity	WO/E	77.63	62.01	78.68	51.45	57.07	65.29	89.56
	W/E	94.04	94.40	76.36	-	-	66.12	94.85
F1 score	WO/E	63.45	79.88	60.96	76.22	33.84	73.82	79.99
	W/E	67.71	81.63	69.43	-	-	75.94	84.59
DSC	WO/E	25.49	40.86	22.84	28.01	26.65	42.33	49.13
	W/E	31.77	52.27	28.20	-	-	58.97	63.52

**Table 4:** 95% confidence intervals for lung stone dataset. Here, WO/E and W/E are without and with the edge conservative module, respectively. Our RenseNet is the combined Rense block with edge conservative module.

Lung Tumor								
Metrics		ResNet50	DenseNet121	EfficientNet [23]	ViT [24]	GAIN [32]	sRense Block	Rense Block
Accuracy	WO/E	0.0124	0.0017	0.0052	0.0039	0.0152	0.0175	0.0076
	W/E	0.0144	0.0078	0.0029			0.0186	0.0208
Precision	WO/E	0.0028	0.0084	0.0070	0.0116	0.0070	0.0174	0.0109
	W/E	0.0084	0.0202	0.0197			0.0149	0.0001
Sensitivity	WO/E	0.0170	0.0146	0.0153	0.0197	0.0061	0.0008	0.0175
	W/E	0.0168	0.0092	0.0160			0.0022	0.0093
Specificity	WO/E	0.0042	0.0018	0.0112	0.0210	0.0171	0.0185	0.0109
	W/E	0.0051	0.0154	0.0202			0.0138	0.0089
F1 score	WO/E	0.0125	0.0101	0.0140	0.0024	0.0204	0.0051	0.0099
	W/E	0.0083	0.0057	0.0159			0.0053	0.0195
DSC	WO/E	2.7190	3.0903	3.8134	1.7193	2.7462	2.2437	1.7087
	W/E	1.6711	2.4934	3.4014	1.9083	3.3200	1.6607	2.9846

**Table 5:** Total number of parameters of the models. Here, WO/E and W/E are without and with the edge conservative module, respectively. Our RenseNet is the combined Rense block with edge conservative module.

		ResNet50	DenseNet121	EfficientNet [23]	ViT [24]	GAIN [32]	sRense Block	Rense Block
Total Number of Parameters [×10^6^]	WO/E	23.51	6.95	63.58	85.64	103.45	0.13	0.15
	W/E	41.91	24.66	91.39	-	-	0.41	0.43

## Data Availability

Individual-level data are not publicly available.
